# Changes in Serum Testosterone After Sublingual Enclomiphene Citrate Combined With a Mineral Oxide Delivery System: A Retrospective Case Series of 15 Men

**DOI:** 10.7759/cureus.109299

**Published:** 2026-05-20

**Authors:** Steven Warren

**Affiliations:** 1 Regenerative Medicine, Regenerative Wellness Center, Salt Lake City, USA

**Keywords:** enclomiphene, hypogonadism, mineral delivery system, non-hormonal therapy, testosterone

## Abstract

Background

Declining testosterone levels in adult males represent a significant clinical burden. Conventional testosterone replacement therapy (TRT) with exogenous androgens suppresses the hypothalamic-pituitary-gonadal (HPG) axis, causes testicular atrophy, and impairs spermatogenesis, outcomes that are unacceptable for many patients. Enclomiphene citrate, a selective estrogen receptor modulator (SERM), stimulates endogenous gonadotropin release and has been studied as an alternative to exogenous TRT.

Objective

This study aims to describe 60-day changes in serum total testosterone in 15 adult men treated with a compounded sublingual formulation, TBooster 365 (Best 365 Labs, Salt Lake City, UT), containing enclomiphene citrate 25 mg, boron 10 mg, vitamin C 10 mg, spermidine 10 mg, and the MODS MAX mineral oxide delivery system per 1 mL dose.

Methods

This is a retrospective case series of 15 consecutive adult male patients with symptomatic hypogonadism, enrolled regardless of baseline total testosterone level, treated at a single private longevity medicine practice located at an elevation of 4,500 to 5,500 feet. All blood draws were fasting morning samples analyzed at reference laboratories by liquid chromatography tandem mass spectrometry (LC-MS/MS) or immunoassay. The primary outcome was the change in total serum testosterone at 60 days.

Results

All 15 patients demonstrated increased total testosterone at 60 days. Mean total testosterone increased from 347.0 ng/dL to 805.0 ng/dL (mean change: +458.0 ng/dL; 95% CI (326.3, 589.6); t(14) = 7.46; p < 0.0001 by paired t-test; Wilcoxon signed-rank W = 0, p = 0.0001). The paired-samples Cohen's dz was 1.93; the pooled-SD Cohen's d was 2.37. The mean body weight change across the cohort was -0.5 lb (range: -40 to +15 lb), with individual patients gaining, losing, or maintaining weight without a standardized diet or exercise protocol. Sensitivity analyses excluding the largest responder (Patient 4, who was also on concurrent GLP-1 receptor agonist therapy with substantial weight loss) and restricting to patients with a body weight change of 10 lb or less both preserved the primary finding (dz 2.35 and 2.31, respectively). No serious adverse events were documented during the 60-day period.

Conclusion

In this retrospective uncontrolled case series of 15 symptomatic men, a compounded sublingual protocol containing enclomiphene citrate, boron, vitamin C, spermidine, and MODS MAX was associated with increased total testosterone after 60 days. Because the study lacked a control group, standardized symptom assessment, luteinizing hormone (LH)/follicle-stimulating hormone (FSH) measurements, and long-term safety monitoring, causality, clinical efficacy, fertility preservation, HPG-axis effects, and the independent contribution of MODS MAX cannot be determined. Prospective controlled studies are needed.

## Introduction

Testosterone deficiency affects an estimated 10-40% of adult men, with population studies documenting a secular decline of approximately 1% per year since the 1980s [1,2]. A large state-mandated health organization study confirmed these trends across age groups, independent of obesity or comorbidity [3]. These declines carry implications for male metabolic health, cardiovascular function, mood, cognitive performance, and fertility, motivating the search for safe, effective, and reversible therapeutic strategies.

Conventional testosterone replacement therapy (TRT) with exogenous androgens restores serum testosterone but suppresses endogenous luteinizing hormone (LH) and follicle-stimulating hormone (FSH) production through negative feedback on the hypothalamic-pituitary-gonadal (HPG) axis [4]. This results in testicular atrophy, impaired spermatogenesis [5], and dependence on lifelong exogenous administration, which are unacceptable outcomes for many patients, particularly younger men and those wishing to preserve fertility [6].

Enclomiphene citrate, the trans-isomer of clomiphene, acts as a selective estrogen receptor modulator (SERM) at the hypothalamus and anterior pituitary, blocking estradiol negative feedback and thereby stimulating endogenous gonadotropin release and downstream testosterone synthesis [7,8]. Unlike standard clomiphene, enclomiphene does not contain the cis-isomer zuclomiphene, which carries weak estrogenic activity, making its receptor pharmacology cleaner and more predictable [9-11]. Published Phase II and Phase III trial data confirm that enclomiphene raises serum testosterone in secondary hypogonadal men while also raising or maintaining LH and FSH [7-9]; however, whether these findings generalize to the broader population of symptomatic men in community practice remains an open question.

This case series reports 60-day outcomes in 15 consecutive symptomatic men treated with a compounded sublingual enclomiphene formulation incorporating the MODS MAX mineral oxide delivery system in a longevity medicine practice setting.

## Materials and methods

Study design and setting

This was a retrospective case series conducted at a single private longevity medicine practice in the Salt Lake City area, located at an elevation of approximately 4,500 to 5,500 feet. Data were extracted retrospectively from the electronic health record system at the Regenerative Wellness Center.

Altitude consideration

Because the clinic is located at elevations between 4,500 and 5,500 feet, baseline and follow-up hematocrit values in this cohort are mildly elevated relative to sea-level reference ranges. This is an expected physiological adaptation to residence at a moderate altitude and is not attributable to the study intervention. One patient (Patient 2) additionally required therapeutic phlebotomies during the study window for tobacco-related polycythemia, which had been recognized prior to study initiation and which was managed independently of the testosterone protocol.

Institutional Review Board (IRB) review

This retrospective case series was reviewed by the HappyMD IRB (Registration No. IRB00015091, IORG No. IORG0012771) and determined to be exempt from ongoing IRB review under 45 CFR 46.104(d)(4)(iii). All patient data were de-identified using the safe harbor method [12] prior to analysis.

Rationale for n=15 sample size

This was a retrospective case series rather than a prospective trial; the sample size was determined by the number of consecutive eligible patients who met the inclusion criteria during the observation window. A sample of 15 is consistent with published case series exploring novel testosterone optimization approaches and is sufficient to generate preliminary effect estimates and identify gross safety signals. The limitation of n=15 is acknowledged prominently, and the study is explicitly presented as hypothesis-generating.

Inclusion and exclusion criteria

This study included adult males aged 18 to 70 years presenting with symptoms of hypogonadism (including fatigue, low libido, erectile dysfunction, loss of muscle mass, mood changes, or cognitive complaints); enrolled regardless of baseline total testosterone level; off any form of exogenous testosterone for at least six months; and enrolled consecutively at presentation. The decision to include patients whose baseline total testosterone was within the laboratory reference range but who remained symptomatic reflects routine longevity medicine practice, where total testosterone alone does not capture the clinical burden that correlates more closely with free testosterone, sex hormone-binding globulin (SHBG), and symptom expression.

The study excluded those with active malignancy, severe hepatic or renal disease, or prior TRT within six months of enrollment.

Patients

A total of 15 consecutive adult male patients presenting with symptomatic testosterone deficiency between July 1, 2025, and September 1, 2025 (mean age 44.7 years, SD 15.1; median 49 years; range 22 to 66 years) were included in the study.

Protocol

All 15 patients received TBooster 365 (Best 365 Labs, Salt Lake City, UT), a compounded sublingual liquid formulation. Each 1 mL dose contained enclomiphene citrate 25 mg, boron 10 mg, vitamin C 10 mg, and spermidine trihydrochloride 10 mg, delivered via the MODS MAX mineral oxide proprietary delivery system. The product was administered as a 1 mL sublingual dose, held under the tongue for 30 to 60 seconds, once daily for 60 days. Bottles dispensed to patients were intentionally unlabeled to mitigate expectation bias (nocebo and placebo effects) that could arise from patients reading ingredient labels. Whether MODS MAX independently contributed to the outcomes cannot be determined without an enclomiphene-only control arm.

Cofactor rationale

Boron has been reported to increase free testosterone and reduce SHBG in short-term supplementation studies in healthy volunteers [13,14]. Spermidine, a naturally occurring polyamine found in all living cells, has demonstrated Leydig cell protective effects against immune-mediated oxidative stress in preclinical models [15], induction of autophagy in cell and animal studies [16,17], and emerging signals for longevity-related benefits in translational research [18]. Boron 10 mg and spermidine trihydrochloride 10 mg were included as cofactors in the TBooster 365 formulation administered to all 15 patients. The independent clinical contributions of these cofactors to the testosterone response observed in this series cannot be determined from this uncontrolled case series and are best characterized as untested co-interventions.

Laboratory methods

All blood draws were performed in the fasting state, in the morning (before 10:00 AM), consistent with Endocrine Society recommendations for testosterone measurement [19]. Over the study period, three reference laboratories were used. One reference laboratory that performed early baseline testing subsequently ceased operations, and its web portal became inaccessible during the revision of this manuscript; paired baseline values for affected patients were recovered from paper laboratory printouts retained in the medical record. All testosterone values reported in this manuscript are total testosterone in nanograms per deciliter (ng/dL). On laboratory reports that returned both total testosterone (ng/dL) and direct free testosterone (pg/mL) on the same panel, only the total testosterone value in ng/dL was abstracted. Free testosterone, calculated free testosterone, and bioavailable testosterone were not used. Estradiol was not routinely measured in male patients at this practice and is not reported. Prostate-specific antigen (PSA) was not ordered in patients under age 50 per longstanding practice policy and is accordingly reported as not applicable (NA) for those patients.

Measurements

Fasting serum total testosterone was measured at baseline and at 60 days. Additional baseline and 60-day measurements included body weight, SHBG (baseline only), hematocrit, PSA (patients age 50 and older), blood pressure, and glycated hemoglobin (HbA1c), where clinically indicated. LH, FSH, semen analysis, and standardized validated symptom scales were not part of this retrospective observation and are acknowledged as limitations.

Statistical analysis

Analyses were performed using Python 3.12 (Python Software Foundation, Wilmington, Delaware, United States), with the SciPy statistical library (SciPy developers, Austin, Texas, United States) used for the paired t-test and related analyses. The normality of the 15 paired change scores was tested by the Shapiro-Wilk test [20]. The paired t-test [21] served as the primary analysis. Effect size is reported as paired-samples Cohen's dz and pooled-SD Cohen's d [22]. Two sensitivity analyses were specified a priori: a non-parametric Wilcoxon signed-rank test [23] and the exclusion of the largest responder. A post-hoc sensitivity analysis excluding a single patient on concurrent GLP-1 receptor agonist therapy with extreme weight loss (Patient 4) was added to address whether the overall finding was robust to the most metabolically atypical patient. A second post-hoc sensitivity analysis restricted the sample to patients with an absolute weight change of 10 lb or less to examine whether the testosterone response was confounded by body composition change.

## Results

Patient demographics

The mean age of the patients was 44.7 years (SD 15.1; median 49; range 22 to 66). The mean baseline total testosterone was 347.0 ng/dL (SD 145.0).

Primary outcome

The mean total testosterone increased from 347.0 plus or minus 145.0 ng/dL at baseline to 805.0 plus or minus 231.5 ng/dL at 60 days. The mean change was +458.0 ng/dL (SD 237.7; 95% CI 326.4 to 589.6 ng/dL; t(14) = 7.46; p < 0.0001 by two-tailed paired t-test), representing a mean increase of approximately 132%. Individual patient results are presented in Table 1.

**Table 1:** Baseline and 60-day total testosterone (n=15). All blood draws were fasting morning samples obtained before 10:00 AM, consistent with Endocrine Society recommendations. Three reference laboratories were used across the study window; baseline values for some patients were recovered from paper laboratory printouts after one laboratory ceased operations. The "Mean" row represents the cohort mean for each column. T = total serum testosterone

Patient	Age (years)	Baseline T (ng/dL)	Follow-up T (ng/dL)	Change (ng/dL)	% Change
1	33	170.2	923.0	+752.8	+442.1
2	51	433.0	868.0	+435.0	+100.5
3	66	520.5	1025.0	+504.5	+96.9
4	32	123.0	1181.0	+1058.0	+860.2
5	55	637.7	1014.7	+377.0	+59.1
6	40	486.8	1016.0	+529.2	+108.7
7	25	408.9	668.5	+259.6	+63.5
8	58	345.0	529.3	+184.3	+53.4
9	64	195.0	658.5	+463.5	+237.7
10	59	297.0	980.0	+683.0	+230.0
11	24	235.0	643.0	+408.0	+173.6
12	22	234.0	467.6	+233.6	+99.8
13	38	345.0	622.0	+277.0	+80.3
14	49	468.9	989.0	+520.1	+110.9
15	55	305.0	489.0	+184.0	+60.3
Mean	44.7	347.0	805.0	+458.0	+132.0

Effect Sizes

The paired-samples Cohen's dz was 1.93. The pooled-SD Cohen's d was 2.37. Both estimates indicate very large effect sizes by conventional standards.

Non-parametric Confirmation

The Wilcoxon signed-rank test yielded W = 0 and p = 0.0001, confirming the primary result.

Sensitivity Analysis Excluding the Largest Responder (Patient 4)

The mean change was +415.1 ng/dL (95% CI (313.2-517.0); t(13) = 8.80; p < 0.0001). Cohen's dz = 2.35. Wilcoxon W = 0, p = 0.0001. The primary finding is robust to the exclusion of the largest responder, who was also the patient with the most pronounced concurrent metabolic and lifestyle changes (GLP-1 receptor agonist, dietary change, and substantial weight loss).

Sensitivity Analysis Restricted to Patients With a Weight Change of 10 lb or less (n = 11)

The mean change was +383.5 ng/dL (95% CI (271.8-495.1); t(10) = 7.65; p < 0.0001). Cohen's dz = 2.31. The testosterone response persisted in patients whose body weight was essentially unchanged, supporting a pharmacologic rather than body-composition-mediated effect.

Lifestyle Confounders in Extreme Responders

Two patients undertook substantial concurrent lifestyle changes during the observation window. Patient 4 (the largest responder, +1058.0 ng/dL) initiated GLP-1 receptor agonist therapy together with a carnivore-style diet and a new resistance-training program, and lost approximately 40 lb during the 60-day window; this single patient is therefore the most heavily co-intervened case in the series. Patient 1 (+752.8 ng/dL) initiated a structured resistance-training program and a comprehensive dietary change, gained approximately 13 lb (consistent with lean-mass accrual), and reported a subjective benefit within the first week of therapy. Both patients are retained in the primary analysis to preserve the consecutive, real-world nature of the case series. The pre-specified sensitivity analyses (reported above) confirm that the overall testosterone response is not dependent on either case: excluding Patient 4 yields +415.1 ng/dL (p<0.0001), and restricting to patients with minimal weight change (which excludes both Patient 4 and Patient 1) yields +383.5 ng/dL (p<0.0001).

**Figure 1:**
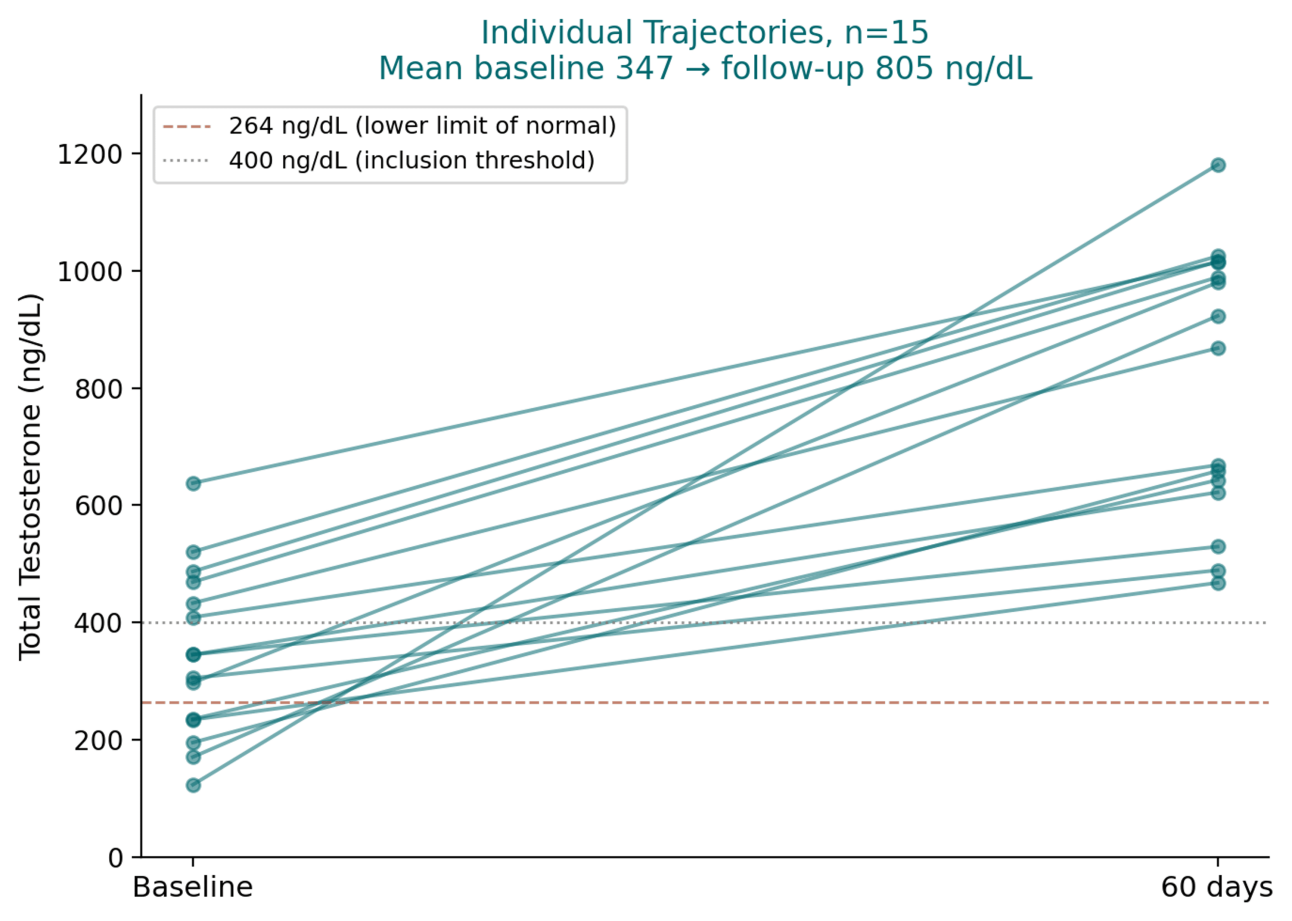
Individual total testosterone trajectories at baseline and 60-day follow-up (n=15). Each line represents one of the 15 male patients, connecting baseline total testosterone (ng/dL) to the value measured after 60 days of sublingual TBooster 365 (enclomiphene citrate 25 mg, boron 10 mg, vitamin C 10 mg, and spermidine trihydrochloride 10 mg, delivered via the MODS MAX mineral oxide system). The dashed red line marks the lower limit of the laboratory reference range (264 ng/dL). The dotted gray line marks the 400 ng/dL reference threshold. The mean baseline 347 ng/dL increased to a mean follow-up value of 805 ng/dL.

**Figure 2:**
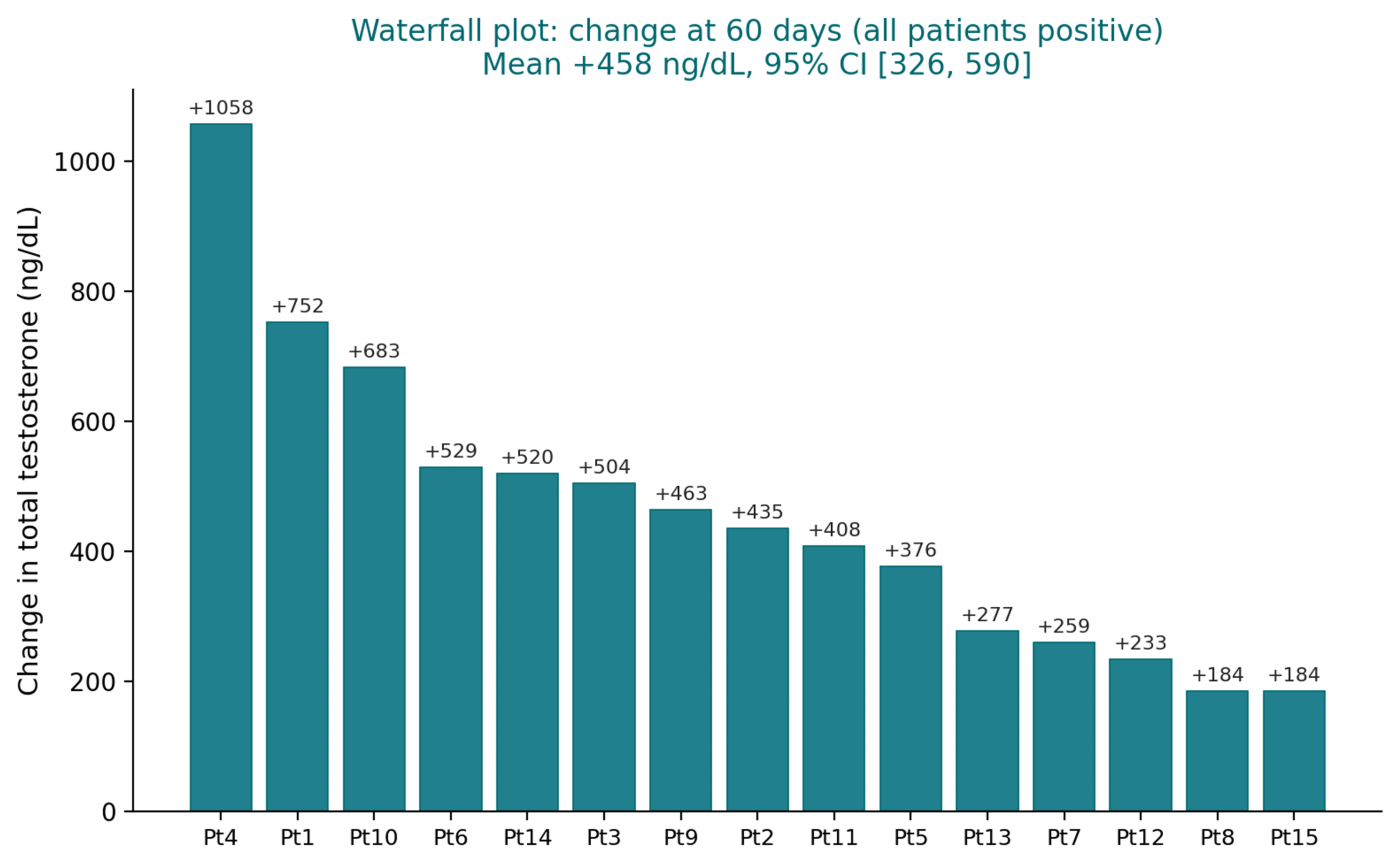
Waterfall plot of absolute change in total testosterone at 60 days (n=15). Each bar represents one patient, sorted from largest to smallest absolute change in total testosterone (ng/dL) from baseline to 60-day follow-up. All 15 patients demonstrated a positive response. The largest individual change was +1058 ng/dL (Patient 4), and the smallest was +184 ng/dL (Patient 15). Mean change: +458 ng/dL (95% CI 326 to 590; p < 0.0001).

All 15 patients demonstrated a positive testosterone change. Individual changes ranged from +184.0 ng/dL (Patient 15) to +1058.0 ng/dL (Patient 4). Trajectories are shown in Figure 1, and individual changes are ranked from largest to smallest in Figure 2.

The magnitude of individual testosterone changes is presented as a waterfall plot, ranking each patient from the largest to the smallest absolute change in total testosterone. All 15 patients demonstrated a positive response, with individual changes ranging from +1058.0 ng/dL (Patient 4) to +184.0 ng/dL (Patient 15) (Figure 2).

Symptoms

All 15 patients (100%) reported subjective improvement in at least one symptom domain at follow-up.

Adverse events

None reported during the 60-day observation period. Secondary metabolic and safety variables, including body weight, SHBG, hematocrit, PSA, blood pressure, and HbA1c at baseline and 60 days, are presented in Table 2.

**Table 2:** Secondary and safety variables (n=15). PSA is reported as "-" for patients under 50 years of age per practice policy. Hematocrit values reflect residence at 4,500 to 5,500 feet elevation and are expected physiological adaptations, not therapy-induced polycythemia. Patient 2 underwent therapeutic phlebotomies during the study window for tobacco-related polycythemia, recognized prior to study initiation and managed independently of the testosterone protocol. Patient 4 initiated GLP-1 receptor agonist therapy and a carnivore-style diet during the study window. Wt B = baseline weight; Wt 60d = 60-day weight; ΔWt = change in weight; SHBG = sex hormone-binding globulin (baseline only); Hct B = baseline hematocrit; Hct 60d = 60-day hematocrit; PSA = prostate-specific antigen; BP = blood pressure; HbA1c = glycated hemoglobin

Patient	Wt B (lb)	Wt 60d (lb)	ΔWt (lb)	SHBG (nmol/L)	Hct B (%)	Hct 60d (%)	PSA B (ng/mL)	PSA 60d (ng/mL)	BP B	BP 60d	HbA1c (%)
1	165	178	+13	14	46	52	-	-	110/80	105/74	5.1
2	205	210	+5	36	56	57	0.80	1.90	115/76	110/82	5.6
3	175	180	+5	37	45	50	0.40	0.80	125/80	126/82	5.4
4	300	260	-40	57	49	50	-	-	130/82	125/72	5.8
5	225	240	+15	38	52	53	1.50	1.80	130/78	120/78	5.1
6	175	178	+3	40	47	48	-	-	120/65	120/68	4.6
7	150	160	+10	19	45	47	-	-	115/70	110/68	5.0
8	160	170	+10	28	49	50	0.80	0.90	115/78	110/75	5.0
9	220	200	-20	50	47	50	1.53	2.00	130/75	130/76	5.0
10	225	220	-5	39	48	52	2.00	2.10	120/78	120/72	5.1
11	175	178	+3	39	46	47	-	-	114/65	115/68	5.0
12	210	200	-10	39	47	49	-	-	115/70	110/68	4.8
13	180	190	+10	30	47	49	-	-	130/76	126/76	5.1
14	240	230	-10	68	46	49	-	-	140/85	138/82	5.2
15	175	178	+3	20	47	49	0.90	1.00	130/78	128/75	5.0

## Discussion

This retrospective case series describes increases in total serum testosterone in 15 symptomatic men treated for 60 days with a compounded sublingual formulation containing enclomiphene citrate, boron, vitamin C, spermidine, and the MODS MAX delivery system. The mean increase of 458.0 ng/dL (approximately 132%) was statistically significant and robust in both pre-specified sensitivity analyses (exclusion of the largest responder, who was also the GLP-1/lifestyle case, and restriction to patients with minimal body weight change).

Attribution

Because no enclomiphene-only control group was included, the independent contribution of MODS MAX, boron, vitamin C, or spermidine cannot be determined. The testosterone response observed is consistent in magnitude with published enclomiphene data [7-9], suggesting that enclomiphene was likely the primary driver. MODS MAX and the co-delivered micronutrients are best characterized as untested co-interventions.

Patient acceptability of a sublingual oral formulation

A repeated qualitative observation in this case series, although not a pre-specified endpoint, was that patients reported a strong preference for a once-daily sublingual liquid over injectable, transdermal cream, or subcutaneous pellet alternatives. Conventional exogenous TRT carries documented adverse effects, including erythrocytosis, sleep apnea exacerbation, and concerns regarding cardiovascular and prostate safety [24]. Beyond pharmacologic adverse effects, injectable testosterone esters introduce peak-and-trough variability in mood and energy and the burden of needle administration; transdermal creams carry the risk of secondary transfer to female partners and children; and subcutaneous pellets carry procedural risks of insertion and extrusion together with fixed pharmacokinetics that cannot be down-titrated after placement. A sublingual SERM-based protocol bypasses each of these issues. While patient acceptability was not formally measured in this retrospective series, the absence of dropouts at 60 days is consistent with this informal observation.

Body composition and pharmacologic effect

A key concern with any retrospective series in which weight change occurs is whether the testosterone response reflects body composition change rather than the study intervention. In this cohort, body weight changed in both directions and across a wide range (minus 40 to plus 15 lb). Patient 4 is an informative case: he began the study at 300 lb, was on a GLP-1 receptor agonist, and lost approximately 40 lb during the 60-day window. His testosterone response was the largest in absolute magnitude in the cohort. When he is excluded, the mean response remains highly significant (dz = 2.35, p < 0.0001). Conversely, when analysis is restricted to patients whose weight changed by 10 lb or less, the testosterone response also remains highly significant (dz = 2.31, p < 0.0001). Together, these sensitivity analyses argue against an explanation in which body composition change is driving the primary result.

Effect size and clinical significance

The mean change moved the group mean from 347 ng/dL to 805 ng/dL, which is well above the lower limit of normal. Whether these biochemical changes translated to durable symptom improvement or functional benefit cannot be established from this dataset.

Regression to the mean

Patients were selected on the basis of meeting a low baseline threshold. Some portion of the observed testosterone increase may reflect regression toward the population mean on repeated measurement. This cannot be distinguished from a true treatment effect without a concurrent control group.

Altitude and hematocrit

Baseline and follow-up hematocrits in this cohort (approximately 45-57%) reflect residence at an elevation of 4,500 to 5,500 feet. This is an expected physiological adaptation, not therapy-induced polycythemia. One patient (Patient 2) required therapeutic phlebotomies during the study window for tobacco-related polycythemia that predated the protocol.

Age and Leydig cell reserve

The two largest responders in this series were among the youngest patients (Patient 1, age 33; Patient 4, age 32). This pattern is biologically plausible: enclomiphene acts by blocking estrogen-mediated negative feedback at the hypothalamus and pituitary, thereby increasing endogenous LH and FSH drive to the testes [7,8]. The magnitude of the downstream testosterone response is therefore constrained by Leydig cell reserve, which is preserved in younger men and progressively diminishes with age. Clinically, men in their 20s and 30s and older men with otherwise intact testicular function who decline injectable therapy tend to be the strongest responders to this non-hormonal approach. This observation is hypothesis-generating and warrants prospective evaluation stratified by age and baseline gonadotropin status.

Limitations

The following are the study's limitations: (1) retrospective, uncontrolled design, where no control group was included and causality cannot be established; (2) small sample size (n=15). The study is underpowered for definitive efficacy or safety conclusions; (3) no LH or FSH measurements. HPG-axis preservation cannot be concluded from this dataset; (4) no semen analysis. Fertility preservation cannot be inferred; (5) multiple reference laboratories. Three reference laboratories were used across the study window; one closed during the study and is no longer accessible. Paired baseline values for some patients were recovered from paper printouts. All reported testosterone values are total testosterone in ng/dL; (6) no standardized diet or exercise protocol. Body weight changed in both directions across a wide range. Sensitivity analyses mitigate but do not eliminate confounding from this source; (7) no validated symptom scale, no baseline severity score. Symptom data, where present, are exploratory only; (8) passive adverse event monitoring. Safety laboratories were not systematically obtained for all patients; (9) single site, single treating physician. Generalizability is limited; (10) short follow-up (60 days). Long-term efficacy, durability, and safety are unknown; (11) regression to the mean. Low-baseline selection may contribute to observed increases; and (12) proprietary delivery system. MODS MAX is proprietary and patent-pending, which limits independent replication of the exact formulation.

## Conclusions

In this retrospective uncontrolled case series of 15 symptomatic men, a compounded sublingual protocol containing enclomiphene citrate, boron, vitamin C, spermidine, and MODS MAX was associated with increased total testosterone after 60 days, with every patient demonstrating a positive response. The result was robust across both pre-specified sensitivity analyses, including exclusion of the largest responder (who was also the patient with concurrent GLP-1 receptor agonist therapy and substantial weight loss) and restriction to patients with minimal body weight change. Because the study lacked a control group, standardized symptom assessment, LH and FSH measurements, semen analyses, and long-term safety monitoring, causality, clinical efficacy, fertility preservation, HPG-axis effects, and the independent contribution of MODS MAX cannot be determined. Prospective controlled studies comparing enclomiphene alone versus enclomiphene plus MODS MAX are needed.
